# Study of Antispasmodic and Antidiarrheal Activities of *Tagetes lucida* (Mexican Tarragon) in Experimental Models and Its Mechanism of Action

**DOI:** 10.1155/2020/7140642

**Published:** 2020-10-28

**Authors:** Rosa Ventura-Martinez, Guadalupe Esther Angeles-Lopez, Maria Eva Gonzalez-Trujano, Omar F. Carrasco, Myrna Deciga-Campos

**Affiliations:** ^**1**^ Departamento de Farmacologia, Facultad de Medicina, Universidad Nacional Autónoma de México (UNAM), Av Universidad No. 3000, Col. Copilco Universidad, Ciudad Universitaria, 04510 Ciudad de México, Mexico; ^2^Laboratorio de Neurofarmacologia de Productos Naturales, Departamento de Investigaciones en Neurociencias, Instituto Nacional de Psiquiatría “Ramón de La Fuente Muñiz”, México-Xochimilco 101, Col. San Lorenzo Huipulco, 14370 Ciudad de México, Mexico; ^3^Seccion de Estudios de Posgrado e Investigacion, Escuela Superior de Medicina, Instituto Politécnico Nacional (IPN), Plan de San Luis y Díaz Mirón s/n, Col. Casco de Santo Tomás, 11340 Ciudad de México, Mexico

## Abstract

*Tagetes lucida* has been used in traditional medicine as a remedy to alleviate several gastrointestinal disorders that provoke stomachaches, abdominal cramps, and diarrhea. However, there is not enough scientific evidence that supports these effects. Therefore, the purpose of this study was to evaluate antispasmodic and antidiarrheal activities of aqueous extract of *T. lucida* (AqExt-TL) as well as its mechanism of action in experimental models. Antispasmodic activity and the mechanism of action of AqExt-TL were assessed on segments of the guinea pig ileum precontracted with KCl, acetylcholine (ACh), or electrical field stimulation (EFS). Furthermore, the antispasmodic effect of two coumarins (umbelliferone and herniarin) previously identified in this species was evaluated. Antidiarrheal activity of AqExt-TL was determined using the charcoal meal test in mice. AqExt-TL showed antispasmodic activity in segments of the guinea pig ileum precontracted with KCl (83.7 ± 1.9%) and ACh (77.2 ± 5.3%) at the maximal concentration; however, practically, it did not alter the contractions induced by EFS (10.1 ± 2.2%). Antispasmodic activity of AqExt-TL was not significantly altered by hexamethonium (a ganglionic blocker) or L-NAME (an inhibitor of nitric oxide synthase). However, this extract decreased the maximal contractile response to calcium (82.7 ± 8.5%), serotonin (68.1 ± 8.5%), and histamine (63.9 ± 5.9%) in their concentration-response curves. Umbelliferone and herniarin also induced an antispasmodic effect on tissues precontracted with KCl. In addition, low doses of AqExt-TL reduced to 50% the distance traveled by charcoal meal in the gastrointestinal transit model in mice as loperamide, an antidiarrheal agent, did. These results provided evidence of the antispasmodic and antidiarrheal activity of *T. lucida*, which supports its use in the folk medicine in relieving symptoms in some gastrointestinal disorders. In the antispasmodic effect, the blockade of histaminergic and serotoninergic pathway as well as the calcium channels seems to be involved. Finally, umbelliferone and herniarin could be partially responsible for the antispasmodic activity induced by *T. lucida*.

## 1. Introduction

Functional gastrointestinal disorders such as dyspepsia or irritable bowel syndrome cause stomachaches, abdominal cramps, and diarrhea. These symptoms have been traditionally treated with herbal medicines all over the world [1, 2]. *Tagetes lucida* Cav. (*T. lucida*), also known as “hierba de Santa María,” “pericón,” “hierbanis,” “periquillo,” “anicillo” or Mexican tarragon, is an endemic plant in Central America and Mexico that is prepared from fresh flowers or from aerial parts of the plant and orally consumed as infusion to alleviate stomachaches and diarrhea, mental agitation, or symptoms of a hangover, as well as infections caused by parasites [3].

Phytochemical studies of our group of work have identified coumaric components in the aqueous extract of this species using thin layer chromatography and Ultra-High-Performance Liquid Chromatography techniques [4]. Other studies have determined the presence of umbelliferone, herniarin, and scoparone among other coumarins [5, 6]; in addition, some flavonoids such as quercetin, patuletin, rutin, isorhamnetin, quercetagetin, and naringenin have been identified [6–8].

Pharmacological studies of *T. lucida* have reported its anxiolytic [4], antibacterial [9, 10], antioxidant [11], cytotoxic [12, 13], insecticidal [14], antidepressive [15, 16], spasmolytic [17, 18], and antinociceptive activities [19].

Despite its use in gastrointestinal complaints in the traditional medicine, the only scientific study that supports its effectiveness in the intestinal smooth muscle was made with the chloroform extract *T. lucida* leaves. Authors reported that the chloroform extract reduced the contractile activity in the jejunum of rabbits [17]. However, although the aqueous infusion is popularly consumed, there is no evidence on the effect of the aqueous extract of *T. lucida* on the gastrointestinal system, let alone on the mechanism of action involved. For this, the purpose of this study was to evaluate the antispasmodic and antidiarrheal effects of aqueous extract of aerial parts of *T. lucida* in two experimental models and to investigate its mechanism of action. We also determined the antispasmodic effect of umbelliferone and herniarin as possible bioactive components of this species.

## 2. Materials and Methods

### 2.1. Plant Material and Preparation of Aqueous Extract of *T. lucida* (AqExt-TL)

The aerial parts of *T. lucida* Cav. (Asteraceae) were collected in Amatlán de Quetzalcóatl, State of Morelos, Mexico. The species identification was confirmed by taxonomist M.S. Abigail Aguilar Contreras. A voucher specimen (IMSSM No.15878) was deposited in the herbarium of the “Instituto Mexicano del Seguro Social” in Mexico City. The aqueous extract of *Tagetes lucida* (AqExt-TL) was obtained using the procedure described by Pérez-Ortega et al. [4]. Briefly, 220 g of dried aerial parts of *T. lucida* was added to 1000 mL of boiling water for 60 minutes. Infusion was filtered and lyophilized using a Labconco instrument. For the *in vitro* experiments, the AqExt-TL was prepared with saline solution (0.9%) before adding it to the organ bath to get 31.6, 100, 316, 562, and 1000 *μ*g/mL. For the *in vivo* experiments, the AqExt-TL was also prepared with saline solution (0.9%) just before its administration.

### 2.2. Animals

Eight adult male guinea pigs (300 g) and thirty adult male CD1 mice (25 g) were obtained from the Central Bioterium of the Faculty of Medicine of Universidad Nacional Autónoma de Mexico (UNAM) to determine the antispasmodic and antidiarrheal effect, respectively. Five days before the experiments, animals were kept in a room with temperature and humidity controlled (22 ± 2°C and 45–60%) with an automatically timed cycle of 12 h light/dark (lights on 7–19 h); they had access to food (Purina Chow, St. Louis, MO, USA) and water *ad libitum* for acclimation. For the *in vitro* experiments, guinea pigs were housed in cages of transparent acrylic of 44 × 34 × 18 cm height (one animal per cage) with removable mesh cover. For the *in vivo* experiments, mice were housed in cages of transparent acrylic of 27 × 18.5 × 12 cm height (six animals per cage) with removable mesh cover. Twelve hours before experiments, food was withheld and free access to water was maintained. All experiments were performed between 8 and 14 h. The protocol was approved by the local Ethics and Research Committees of the Faculty of Medicine (FM/DI/054/2018). The experimental protocol was developed following the provisions of the Declaration of Helsinki and recommendations of the Official Mexican Norm for Animal Care and Management [20].

### 2.3. Drugs and Chemicals

Acetylcholine chloride (ACh), papaverine hydrochloride, hexamethonium chloride, *N*_*ω*_-nitro-L-arginine methyl ester hydrochloride (L-NAME), histamine dihydrochloride, serotonin hydrochloride (5-hydroxytryptamine or 5-HT), pyrilamine maleate, ketanserin (+) tartrate, verapamil hydrochloride, umbelliferone (7-hydroxycoumarin), herniarin (7-methoxycoumarin), and loperamide hydrochloride were obtained from Sigma (ST. Louis, Mo, USA). Calcium chloride (CaCl_2_) and potassium chloride (KCl) were bought from J.T. Baker. All chemicals were dissolved or suspended in saline solution (0.9%) and prepared on the day of the experiments. Saline solution was used as vehicle (VEH).

### 2.4. Isolated Guinea Pig Ileum Preparations (*In Vitro* Model)

Antispasmodic activity of AqExt-TL was studied using isolated tissues from guinea pig ileum (*in vitro* experiments). The experiments were performed using ileum sections taken from eight guinea pigs under the procedure described by Ventura-Martinez et al. [21]. Briefly, guinea pigs were sacrificed by cervical dislocation. Approximately 15 cm of ileum was cut of each animal and placed in Krebs-bicarbonate solution (KBS) at 37°C with 95% O_2_ and 5% CO_2_. The KBS had the following composition: 1.2 mM MgCl_2_, 1.2 mM NaH_2_PO_4_, 4.7 mM KCl, 2.5 mM CaCl_2_, 25 mM NaHCO_3_, 118 mM NaCl, 11 mM glucose, and 0.3 mM choline chloride. Six segments of 2 cm were obtained of each guinea pig's ileum and placed independently in organ‐baths containing KBS at 37°C with 95% O_2_ and 5% CO_2_. Segments were equilibrated for 60 minutes under 1 g resting tension. Ileum contractions were measured with force displacement transducers (Grass FT-03C) connected to a polygraph (Grass 7B) with a computerized system of data acquisition (PolyView system, version 2.5).

#### 2.4.1. Antispasmodic Effect of AqExt-TL on the Guinea Pig Ileum

After the period of stabilization, the antispasmodic activity of AqExt-TL or papaverine (30 *μ*M), used as positive control, was assessed in six ileum segments precontracted with KCl (32 mM). Once the maximal contraction induced by KCl was reached, the corresponding concentration of AqExt-TL was added to the organ bath and remained in contact with the ileum segments for at least 5 minutes or until the relaxing effect obtained was maintained for at least 10 seconds. This procedure was repeated for each concentration of AqExt-TL (from 31.6–1000 *μ*g/mL) and papaverine (30 *μ*M). Between each concentration, the ileum segments were rinsed with 30–60 mL of warm KBS and were allowed to stabilize for 20 minutes, sufficient time to recover their spontaneous activity [22]. Concentrations of AqExt-TL were selected following logarithmic increments to cover the (CRC).

In a second set of experiments, other six ileum segments were contracted with electrical field stimulation (EFS) through two nickel electrodes parallelly positioned to the preparation and connected to a stimulator (Grass S88). The following parameters were used to produce the maximal contractile response: 0.3 Hz of frequency, 3 ms of duration, and 14  V of intensity. The ileum segments electrically stimulated were exposed by separation of cumulative concentrations of the AqExt-TL (from 31.6–1000 *μ*g/mL). This procedure was repeated for papaverine (30 *μ*M) [23]. Between AqExt-TL and papaverine, the ileum segments were rinsed with 30–60 mL of warm KBS and were allowed to stabilize for 20 minutes.

The diminution of the contraction induced by KCl or electrical field stimulation (EFS) with each treatment was considered as antispasmodic activity and was compared with the effect induced by saline solution (VEH). The half maximal effective concentration (EC_50_) of AqExt-TL on each experimental protocol was obtained.

In the other six ileum segments, cumulative (CRCs) from 1 × 10^−9^–1 × 10^−5^ M to ACh were obtained in the presence of AqExt-TL (from 31.6–1000 *μ*g/mL) or papaverine (30 *μ*M), incubated for 20 minutes beforehand. Seven CDRs were obtained for each preparation. The ileum segments were rinsed with 30–60 mL of warm KBS and equilibrated by 20 minutes between each CRC. The effect of AqExt-TL or papaverine on contractile response induced by ACh was calculated with the maximal contractile response (*E*_max_) obtained with ACh without treatment, which was considered 100% [24]. Also, the EC_50_ values of ACh with VEH or with each treatment were compared.

#### 2.4.2. Determination of the Mechanism of Action of the Antispasmodic Effect of AqExt-TL on the Guinea Pig Ileum

The mechanism of the antispasmodic activity of AqExt-TL was investigated by exploring the participation of the nitric oxide (NO) pathway, nicotinic receptors, calcium channels, and serotoninergic or histaminergic pathway. To explore the participation of NO pathway or nicotinic receptors in the antispasmodic activity of *T. lucida*, six ileum segments were used. First, the ileum segments were incubated for 30 minutes with VEH (saline solution) or L-NAME (100 *μ*M), an inhibitor of the NO synthase after obtaining a maximal contractile response with KCl (32 mM). After, the previously calculated EC_50_ of AqExt-TL was added. This procedure was repeated but with the ileum segments incubated for 10 minutes with a ganglion blocker, hexamethonium (0.5 mM). The preparations were rinsed with 30–60 mL of warm KBS and were allowed to stabilize for 20 minutes before hexamethonium [21].

An independent group of six ileum segments were used to determine the involvement of the channel's calcium in the antispasmodic activity of *T. lucida.* For this, the segments were stabilized in Ca^+2^-free KBS, and the CRCs to CaCl_2_ (0.05–25 mM) were constructed in the absence or presence of AqExt-TL (31.6–1000 *μ*g/mL) or a calcium channel blocker, verapamil (0.01 *μ*M) [25]. Finally, to explore the participation of serotoninergic or histaminergic pathway, CRCs to serotonin (5-HT) or histamine were constructed in other six ileum segments in the absence or presence of the previously calculated EC_50_ of AqExt-TL, ketanserin (2.5 *μ*M, a 5-HT_2A_ antagonist), or pyrilamine (3 *μ*M, an H_1_ antagonist) [26]. As in the previous protocols, preparations were rinsed with 30–60 mL of warm KBS and equilibrated for 20 minutes between each CRC. The maximal response obtained with serotonin, histamine, or CaCl_2_, from their respective cumulative CRCs (in the absence of any treatment, VEH), was taken as 100% of the contractile response.

#### 2.4.3. Determination of Antispasmodic Activity of Umbelliferone and Herniarin on the Guinea Pig Ileum

Antispasmodic activity of umbelliferone (1–316 *μ*g/mL) and herniarin (1–316 *μ*g/mL) was assessed on independent groups of ileum segments (six per group) precontracted with KCl (32 mM). As in the previous protocols, preparations were rinsed with 30–60 mL of warm KBS and equilibrated for 20 minutes between every concentration of each coumarin. The diminution of the contraction induced by KCl with each treatment was considered as antispasmodic activity. Concentrations of each coumarin were selected following logarithmic increments to cover the concentration-response curve (CRC). The EC_50_ of each coumarin was obtained.

### 2.5. Effect of AqExt-TL on the Gastrointestinal Transit in Mice (*In Vivo* Model)

The effect of AqExt-TL on the intestinal motility was determined using the charcoal meal test in mice (*in vivo* experiment) described by Khan et al. [27]. On day of the experiment, AqExt-TL (31.6, 100, or 316 mg/kg), loperamide (LOP 5 mg/kg, as the positive control), or vehicle (saline solution 0.9%) was orally administered to different groups of mice (6 animals per group). After thirty minutes, 0.3 mL of suspension of charcoal meal (10% charcoal in 5% gum Arabic) was given orally to the animals. After twenty minutes, mice were euthanized by cervical dislocation and the whole small intestines of animals were extracted from pylorus to the caecum. The distance between the pylorus region and the front of the charcoal meal as well the length of the small intestine was measured to obtain the percentage of gastrointestinal motility with each treatment. Doses of AqExt-TL were selected following logarithmic increments.

### 2.6. Statistical Analysis

For the *in vitro* experiments, the effects of each treatment were analyzed using six ileum segments in each experimental protocol, and the results are expressed as the mean ± standard error of the mean (SEM). Statistical differences between treatments were determined using analysis of variance (ANOVA) by repeated measures followed by Dunnett's post hoc test in comparison to the effect of VEH in the corresponding protocol. Statistical differences between *E*_max_ and EC_50_ of umbelliferone and herniarin in their CRC were determined using Student's test. For the *in vivo* experiments, the effects of each treatment on the gastrointestinal motility were analyzed using six mice for each treatment, and the results are expressed as the mean ± SEM. Statistical differences between treatments were determined using one-way ANOVA followed by Dunnett's post hoc test in comparison to the effect of VEH. Statistical analysis was done with GraphPad Prism version 6 for Windows (GraphPad Software, San Diego, CA, USA). In all cases, data follow a normal distribution, and post hoc test was used only if F achieved *P* < 0.05 and there was no significant variance in homogeneity. Differences between means with a *P* < 0.05 were considered significant at the 95% confidence level.

## 3. Results

Regarding the preparation of AqExt-TL, the dry weight yield of the final crude aqueous extract was 8.94 g, which represented 4.08% of the vegetal material.

### 3.1. Antispasmodic Effect of AqExt-TL on the Guinea Pig Ileum and the Analysis of Its Mechanism of Action

**Figure 1:**
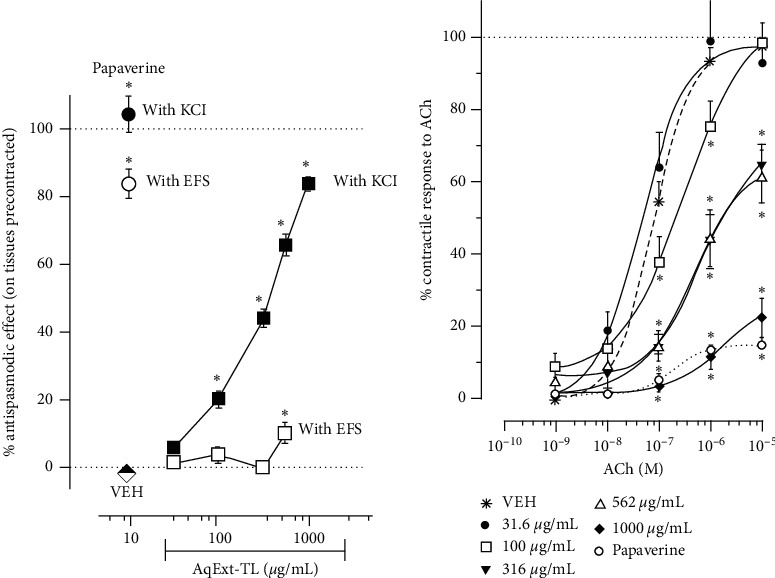
(a) Antispasmodic effect induced by AqExt-TL (31.6–1000 *μ*g/mL) or papaverine (30 *μ*M) on the guinea pig ileum segments precontracted with KCl (32 mM) or EFS. (b) CRCs of ACh in the absence and presence of AqExt-TL (31.6–1000 *μ*g/mL) or papaverine (30 *μ*M) on the guinea pig ileum segments. In all cases papaverine was used as positive control. Each point represents the mean ± SEM of 6 experiments in each experimental protocol. ^*∗*^*P* < 0.05, repeated measures ANOVA followed by Dunnett's test vs vehicle (VEH) group.

AqExt-TL diminished the contractile response obtained with KCl on the guinea pig ileum in a concentration-dependent manner from 100 *μ*g/mL (at 79.7 ± 2.4%) in comparison to vehicle, reaching its maximal diminution at 1000 *μ*g/mL (at 16.3 ± 1.9%). Under these circumstances, the EC_50_ of the antispasmodic effect of AqExt-TL was 300 ± 27.8 *μ*g/mL with an *E*_max_ of 83.7 ± 1.9% of relaxation. The contractile response induced by EFS on the guinea pig ileum was significantly reduced only with 562 *μ*g/mL of AqExt-TL (at 89.9 ± 3.3%). In both cases, papaverine (30 *μ*M), the positive control, decreased almost completely the contractile effect induced by KCl or EFS on the guinea pig ileum (Figure 1(a)). Moreover, AqExt-TL shifted the CRC of ACh to the right in a concentration-dependent manner and increased the EC_50_ of ACh at 30.0 ± 2.4 × 10^−6^ M, 64.5 ± 2.8 × 10^−5^, and 55.2 ± 4.5 × 10^−5^ M with 100, 316, and 562 *μ*g/mL of the extract, respectively, in comparison with VEH (7.7 ± 0.1 × 10^−7^ M). The maximal contraction to 1 × 10^−5^ M of ACh on the guinea pig ileum was reduced in presence of 316, 562, and 1000 *μ*g/mL of the AqExt-TL at 65.1 ± 5.2, 61.5 ± 7.4, and 22.8 ± 5.3%, respectively. In fact, the highest concentration of AqExt-TL (1000 *μ*g/mL) produced the higher inhibitory effect on ACh-induced contraction, as papaverine did (Figure 1(b)).

**Figure 2:**
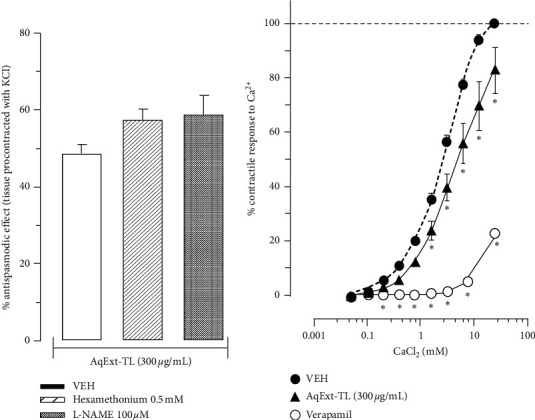
(a) Antispasmodic effect induced by AqExt-TL (300 *μ*g/mL) on the guinea pig ileum segments precontracted with KCl in the absence or in presence of hexamethonium (0.5 mM) or L-NAME (100 *μ*M). Each bar represents the mean ± SEM of 6 experiments. (b) CRCs of CaCl_2_ in the absence and presence of AqExt-TL (300 *μ*g/mL) or verapamil (0.1 *μ*M) on the guinea pig ileum segments. Each point represents the mean ± SEM of 6 experiments. ^*∗*^*P* < 0.05, repeated measures ANOVA followed by Dunnett's test vs vehicle (VEH) group.

**Figure 3:**
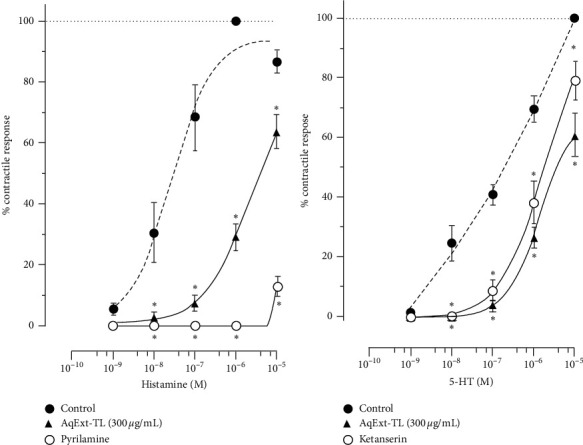
CRCs of the contractile effect of (a), histamine and (b) serotonin (5.HT) in the absence and presence of AqExt-TL (300 *μ*g/mL) and pyrilamine (3 *μ*M) or ketanserin (2.5 *μ*M), their respective antagonists, on the guinea pig ileum segments. Each point represents the mean ± SEM of 6 experiments. ^*∗*^*P* < 0.05, repeated measures ANOVA followed by Dunnett's test vs control group.

In the experiments to explore the mechanism of action, the results showed that neither L-NAME nor hexamethonium significantly modified the antispasmodic effect induced by 300 *μ*g/mL of AqExt-TL on tissues contracted with KCl (Figure 2(a)). However, the same concentration of AqExt-TL reduced the contractile response to CaCl_2_ at 82.7 ± 8.5% at 25 mM, as verapamil did (at 22.6 ± 1.9%), but with much less intensity (Figure 2(b)); even though the extract did not displace the CRC to calcium, it did not change its EC_50_ either in comparison with the VEH group (4.5 ± 1.4 vs 2.8 ± 1.05 mM). Finally, the AqExt-TL caused a rightward shift of both histamine (Figure 3(a)) and 5-HT (Figure 3(b)) CRCs with diminution of the maximum response to each agonist, like that caused by their antagonists (pyrilamine and ketanserin, respectively).

### 3.2. Antispasmodic Effect of Umbelliferone and Herniarin on the Guinea Pig Ileum

**Figure 4:**
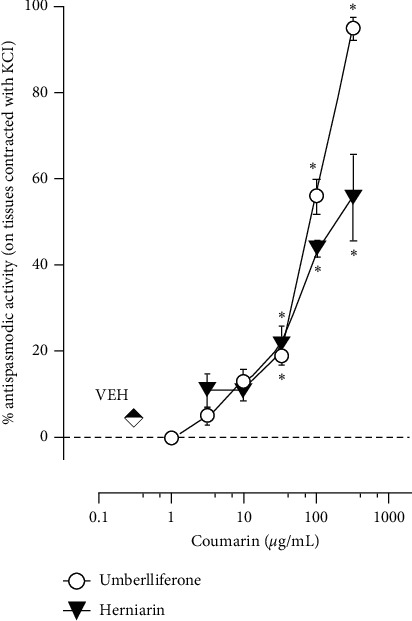
Antispasmodic activity of umbelliferone (1–316 *μ*g/mL) and herniarin (3.16–316 *μ*g/mL) on guinea pig ileum precontracted with KCl. Each point value represents the mean ± SEM of 6 experiments. ^*∗*^*P* < 0.05, repeated measures ANOVA followed by Dunnett's test vs vehicle (VEH) group.

Antispasmodic effects of umbelliferone and herniarin, two coumarins, were also determined in this study. Results showed that umbelliferone and herniarin induced an antispasmodic effect on the guinea pig ileum precontracted with KCl from 31.6 to 316.2 *μ*g/mL with an *E*_max_ of 95.2 ± 3.0% and 55.7 ± 10.1% and an EC_50_ of 75.2 ± 1.1 and 160.1 ± 1.2 *μ*g/mL, respectively (Figure 4). The *E*_max_ and EC_50_ values of both coumarins showed significant difference and, according to these, umbelliferone was more effective and potent than herniarin in inducing an antispasmodic effect.

### 3.3. Effect of AqExt-TL on Gastrointestinal Transit in Mice

**Figure 5:**
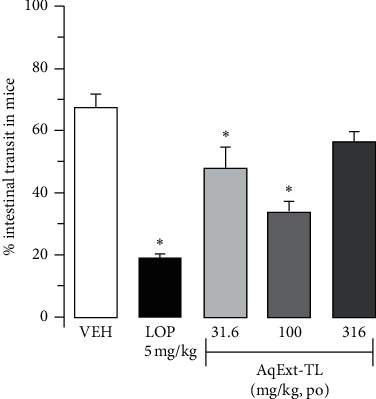
Effect of AqExt-TL (31.6–316 mg/kg, po) or loperamide (LOP, 5 mg/kg, po) on the gastrointestinal transit in mice on the travel of charcoal meal trough small intestine of mice. Each bar represents the mean ± SEM of 6 animals. ^*∗*^*P* < 0.05, one-way ANOVA followed by Dunnett's test vs vehicle (VEH) group.

Finally, oral administration of low doses of AqExt-TL (31.6 and 100 mg/kg) in mice diminished the distance traveled of the charcoal through the small intestine in comparison to that of the VEH group (47.4 ± 7.3, 33.4 ± 3.8 vs 67.0 ± 4.8%, respectively), as loperamide did (18.7 ± 1.5%), an opioid agonist used as antidiarrheal (Figure 5).

## 4. Discussion

*Tagetes lucida* Cav. is used in traditional medicine to treat several gastrointestinal affections in Mexico, Guatemala [28], and Egypt [29]. Even though it is consumed as aqueous infusion, there is low evidence on the effect of the aqueous extract of *T. lucida* on the gastrointestinal system. In this study, the antispasmodic and antidiarrheal effects of AqExt-TL were demonstrated using *in vitro* (guinea pig ileum) and *in vivo* (charcoal meal gut travel distance in mice) models, respectively.

The maximum antispasmodic effect obtained with the AqExt-TL on the guinea pig ileum was independent of tissue relaxation capacity since papaverine (a positive control) completely relaxed tissues precontracted. Papaverine causes relaxation of smooth muscle by inhibition of the cyclic adenosine monophosphate (cAMP) phosphodiesterase, which results in an increase in intracellular cAMP. This event leads to a decrease in the myosin light chain phosphorylation and to an increase in actin depolymerization, thus preventing contraction. On the other hand, papaverine increases Ca^2+^ uptake by the sarcoplasmic reticulum membrane of smooth muscle cells and consequently diminishes the intracellular calcium [30]. It has been traditionally used as reference smooth muscle relaxant to compare spasmolytic or antispasmodic efficacy of several treatments [21, 22].

The antispasmodic effect of AqExt-TL obtained in this study is in agreement with the spasmolytic effect previously reported on the uterine contractility in rats [18]. Also, this effect could be related to the antinociceptive effect recently reported in a visceral pain model in mice, where a hydroalcoholic extract of *T. lucida* reduced contortions produced by acetic acid in mice [19]. Altogether, these results support its use in traditional medicine, where an infusion prepared with this species is used for stomach pain and diarrhea [2].

It is known that contractions induced by high concentrations of KCl are due to an increase in intracellular calcium by opening of L-type Ca^2+^ voltage-dependent channels, which leads to the activation of the myosin light chain on the smooth muscle [30, 31]. Meanwhile, ACh induces contraction by activation of muscarinic receptors (M_3_) on the membrane of smooth muscle and the activation of phospholipase C. This generates inositol triphosphate (IP_3_) that stimulates calcium release from the sarcoplasmic reticulum and induces an increase in the cytosolic levels of calcium [32]. Finally, the contractile effect of EFS is due to a release of ACh from myenteric neurons and its consequent bounding to M_3_ receptors on the membrane of smooth muscle [33]. Our results suggest that the antispasmodic effect of *T. lucida* does not involve the release of ACh from myenteric neurons rather than a direct effect on smooth muscle cells. It was confirmed when the antispasmodic effect of *T. lucida* was not altered by hexamethonium, a ganglion blocker that inhibits release of ACh of the cholinergic terminals in enteric neurons [33]; this effect is not related to neurotransmitter release.

Furthermore, the possible participation of nitric oxide (NO) pathway in the antispasmodic effect of *T. lucida* was investigated; for example, NO is considered a major inhibitory nonadrenergic and noncholinergic neurotransmitter in the gastrointestinal tract that induces relaxation of smooth muscle cells [34, 35]. NO is synthesized from L-arginine by activation of NO synthase, and L-NAME, an inhibitor of NO synthase, inhibits the synthesis of this neurotransmitter. In this study, the antispasmodic effect induced by AqExt-TL was not significantly modified by L-NAME, suggesting that the NO pathway is not involved in this effect.

In addition, antispasmodic activity of *T. lucida* could partially involve a blocking of voltage-dependent calcium channels, since the AqExt-TL decreased the contractile response to calcium, like verapamil did (a calcium channel blocker), but with much less intensity. This protocol has been used in several studies to determine the participation of calcium channels in the relaxing activity of several extracts [22, 36]. Other neurotransmitters, as histamine and serotonin, could be involved in contractions of smooth muscle cells, and compounds that blocked their receptors could inhibit the contractile effect of these endogenous agonists [37]. In this study, the AqExt-TL caused a rightward shift of both histamine and 5-HT CRCs with diminution of the maximum response to each agonist, like that caused by their respective antagonists (pyrilamine for H_1_ receptors and ketanserin for 5-TH_2A_ receptors) suggesting that part of the antispasmodic effect induced by AqExt-TL involves an antagonism on these receptors or a diminution in the release of these neurotransmitters from myenteric neurons.

On the other hand, our results showed that both umbelliferone and herniarin, two coumarins previously identified from the aerial parts of *T. lucida* [5, 6], induced an antispasmodic effect on the guinea pig ileum; this suggests that both coumarins could be involved in the antispasmodic effect induced by this specie. This is not the first study where coumarins seem to be related to a relaxant effect. Oliveira [38] reported the spasmolytic effect of scopoletin, a coumarin isolated from *Brunfelsia hopeana*, but this is the first report on the spasmolytic effect of umbelliferone and herniarin. In fact, the antispasmodic effect of these compounds could be related to the antinociceptive effect recently reported on *T. lucida* in a visceral model pain, where one coumarin seems to be responsible for its antinociceptive effect [39]. Furthermore, other studies have related the biological effects of *T. lucida* to the presence of several coumarins such as esculetin, umbelliferone, scopoletin, 7-8-dihydroxycoumarin, and 6-hydroxy-7-methoxycoumarin [7].

Several flavonoids have been also identified in polar extracts of *T. lucida* such as patuletin, isorhamnetin, quercetin, rutin, quercetagetin, and naringenin [6–8]. In fact, in a previous report of our group of investigation, quercetin and rutin found in another species of *Tagetes*, *Tagetes erecta*, showed spasmolytic activity [40]. The presence of quercetin in extracts of *T. lucida* suggests that it also could be one of the metabolites involved in its antispasmodic effect; previous reports indicate that the spasmolytic effect of quercetin is due to a blockade of voltage-dependent calcium channels of the guinea pig ileum [41, 42], a mechanism studied in this work that seems to be involved in the antispasmodic effect of AqExt-TL.

Finally, the relaxation of the gastrointestinal tract shown by AqExt-TL was according to the inhibition of the propulsion of the charcoal meal in mice due to a reduction in gastrointestinal motility, as our results show. This inhibition of charcoal meal gut travel distance in healthy mice induced by AqExt-TL was similar to the effect induced by loperamide, an antidiarrheal drug. Loperamide, an opiate analogue, is a widely used as an antidiarrheal agent that binds to the opioid receptor in the gut wall [43]. This results in inhibition of ACh and prostaglandin release from myenteric neurons, reducing propulsion peristalsis [43]. The reduction in the intestinal motility induced by *T. lucida* could be related to its antidiarrheal effects reported in traditional medicine [2]. However, it is necessary to do more experiments in other *in vivo* models to demonstrate this activity as well as the mechanism involved.

Data from this study give evidence of some bioactive metabolites of *T. lucida* as partially responsible for its medicinal properties in gastrointestinal affections. It is known that a standardized herbal extract provides a specific concentration of one or more chemical compounds. This fact is important since a specific bioactive metabolite can be characterized, which sometimes allows also knowing targets involved in a selective mechanism of action. A disadvantage of standardization is that it may concentrate one constituent at the expense of other potentially important ones changing the natural balance of the plant's components. In contrast, a full-spectrum extract, like the one used in this study, includes all the plants' chemical or bioactive compounds that may likely attribute the pharmacological activity by interactions between constituents that might be not fully explored. They promote phytochemical arrays with internal complexity working together. It is likely the most common condition in the effect of medicinal plants use in folk medicine.

## 5. Conclusions

In summary, our study provides evidence that the aqueous extract of *T. lucida* aerial parts possesses antispasmodic activity mediated through an antagonism on histaminergic and serotoninergic receptors as well as blocking calcium channels, but it does not involve nicotinic receptors or nitric oxide pathway. This, together with the decrease in intestinal motility induced by the extract in mice, supports the use of *T. lucida* aerial parts in traditional Mexican medicine to treat colic and diarrhea involved in several gastrointestinal disorders. Umbelliferone and herniarin were bioactive compounds partially involved in the antispasmodic effect induced by the aqueous extract of *T. lucida*.

## Data Availability

The data used to support the findings of this study are available from the corresponding author upon request.

## Abbreviations

ACh: Acetylcholine
ANOVA: Analysis of variance
AqExt-TL: Aqueous extract of *T. lucida*
CaCl_2_: Calcium chloride
CRC: Concentration-response curve
EFS: Electrical field stimulation
EC_50_: Half maximal effective concentration
KBS: Krebs-bicarbonate solution
LOP: Loperamide
NO: Nitric oxide
KCl: Potassium chloride
SEM: Standard error of the mean
*E*_max_: The maximal contractile response
VEH: Vehicle.

## References

[1] Kelber O., Bauer R., Kubelka W. (2017). Phytotherapy in functional gastrointestinal disorders. *Digestive Diseases*.

[2] Sayuk G. S., Gyawali C. P. (2015). Irritable bowel syndrome: modern concepts and management options. *The American Journal of Medicine*.

[3] Argueta A., Zolla C., Mata S. (February 2020). Biblioteca digital de la Medicina tradicional mexicana. http://www.medicinatradicionalmexicana.unam.mx/.2009.

[4] Pérez-Ortega G., González-Trujano M. E., Ángeles-López G. E., Brindis F., Vibrans H., Reyes-Chilpa R. (2016). *Tagetes lucida* cav.: ethnobotany, phytochemistry and pharmacology of its tranquilizing properties. *Journal of Ethnopharmacology*.

[5] Ríos T., Flores M. R. (1976). Chemical study of *Tagetes L*. *Revista Latinoamericana de Química*.

[6] Céspedes C. L., Avila J. G., Martínez A., Serrato B., Calderón-Mugica J. C., Salgado-Garciglia R. (2006). Antifungal and antibacterial activities of Mexican tarragon (*Tagetes lucida*). *Journal of Agricultural and Food Chemistry*.

[7] Abdala L. R. (1999). Flavonoids of the aerial parts from *Tagetes lucida* (*Asteraceae*). *Biochemical Systematics and Ecology*.

[8] Xu L. W., Chen J., Qi H. Y., Shi Y. P. (2012). Phytochemicals and their biological activities of plants in *Tagetes L*. *Chinese Herbal Medicines*.

[9] Cáceres A., Figueroa L., Taracena A. M., Samayoa B. (1993). Plants used in Guatemala for the treatment of respiratory diseases. 2: evaluation of activity of 16 plants against Gram-positive bacteria. *Journal of Ethnopharmacology*.

[10] Castillo-Juárez I., González V., Jaime-Aguilar H. (2009). Anti-*Helicobacter pylori* activity of plants used in Mexican traditional medicine for gastrointestinal disorders. *Journal of Ethnopharmacology*.

[11] Aquino R., Cáceres A., Morelli S., Rastrelli L. (2002). An extract of *Tageteslucida* and its phenolic constituents as antioxidants. *Journal of Natural Products*.

[12] Vega-Ávila E., Espejo-Serna A., Alarcón-Aguilar F., Velasco-Lezama R. (2009). Cytotoxic activity of four Mexican medicinal plants. *Proceedings of the Western Pharmacology Society*.

[13] Mejía-Barajas J. A., Del Río R. E. N., Martínez-Muñoz R. E., Flores-García A., Martínez-Pacheco M. M. (2012). Cytotoxic activity in *Tagetes lucida* cav. *Emirates Journal of Food and Agriculture*.

[14] Vera S. S., Zambrano D. F., Méndez-Sanchez S. C., Rodríguez-Sanabria F., Stashenko E. E., Duque Luna J. E. (2014). Essential oils with insecticidal activity against larvae of *Aedes aegypti* (Diptera: Culicidae). *Parasitology Research*.

[15] Guadarrama-Cruz G., Alarcon-Aguilar F. J., Lezama-Velasco R., Vazquez-Palacios G., Bonilla-Jaime H. (2008). Antidepressant-like effects of *Tagetes lucida* Cav. in the forced swimming test. *Journal of Ethnopharmacology*.

[16] Bonilla-Jaime G., Alarcón-Aguilar F., Vega-Avila E., Vazquez-Palacios G., Bonilla-Jaime H. (2012). Antidepressant-like effect of *Tagetes lucida* Cav. Extract in rats: involvement of the serotonergic system. *The American Journal Chinese Medicine*.

[17] López F., Jiménez B., Cortés A., Aoki K. (1990). *Tagetes lucida* Cav I: inhibitory effect on smooth muscle contractility. *Phyton*.

[18] Jayme V., Cortés A., Aoki K. (1998). Effect on rat uterus contractility of *Tagetes lucida* Cav leaf extracts. *Phyton*.

[19] Gutiérrez Gaitén Y. I., Scull Lizama R., García Simón G., Montes Álvarez A. (2018). Evaluación farmacognóstica, fitoquímica y biológica de un extracto hidroalcohólico de *Tagetes lucida* Cavanilles. *Revista Cubana de Plantas Medicinales*.

[20] NOM-062-ZOO-1999 (December 2019). Norma Oficial Mexicana. Secretaría de salud. Especificaciones técnicas para la producción, cuidado y uso de los animales de laboratorio. https://www.gob.mx/senasica/documentos/nom-062-zoo-1999.

[21] Ventura-Martínez R., Rivero-Osorno O., Gómez C., González-Trujano M. E. (2011). Spasmolytic activity of *Rosmarinus officinalis* L. involves calcium channels in the Guinea pig ileum. *Journal of Ethnopharmacology*.

[22] Ventura-Martínez R., Rodríguez R., González-Trujano M. E., Ángeles-López G. E., Déciga-Campos M., Gómez C. (2017). Spasmogenic and spasmolytic activities of *Agastache mexicana* ssp*. mexicana* and *A. mexicana* ssp*. xolocotziana* methanolic extracts on the Guinea pig ileum. *Journal of Ethnopharmacology*.

[23] Teague B., Asiedu S., Moore P. K. (2002). The smooth muscle relaxant effect of hydrogen sulphide in vitro : evidence for a physiological role to control intestinal contractility. *British Journal of Pharmacology*.

[24] Mehmood M. H., Siddiqi H. S., Gilani A. H. (2011). The antidiarrheal and spasmolytic activities of *Phyllanthus emblica* are mediated through dual blockade of muscarinic receptors and Ca^2+^ channels. *Journal of Ethnopharmacology*.

[25] Magalhães P. J. C., Lahlou S., Leal-Cardoso J. H. (2004). Antispasmodic effects of the essential oil of *Croton nepetaefolius* on Guinea-pig ileum: a myogenic activity. *Fundamental and Clinical Pharmacology*.

[26] Cotrim D. M., Figueiredo V. I., Baptista T., Fontes Ribeiro C. A. (2008). Inhibition of serotonin-induced contractions of Guinea pig ileum by *Tilia europeae L*. aqueous extract. *Experimental Pathology and Health Sciences*.

[27] Khan A., Rehman N., Al-Taweel A. M., Perveen S., Fawzy G. A., Gilani A. H. (2012). Studies on prokinetic, laxative, antidiarrheal and gut modulatory activities of the aqueous-methanol extract of *Celtis africana* and underlying mechanisms. *International Journal of Pharmacology*.

[28] Caceres A., Cano O., Samayoa B., Aguilar L. (1990). Plants used in Guatemala for the treatment of gastrointestinal disorders. 1. Screening of 84 plants against enterobacteria. *Journal of Ethnopharmacology*.

[29] Elsayed A. O., Saber F. H., Azza M. N. E. (2015). Some biological activities of *Tagetes lucida* plant cultivated in Egypt. *Advances in Environmental Biology*.

[30] Karaki H., Ozaki H., Hori M. (1997). Calcium movements, distribution and function in smooth muscle. *Pharmacology Reviews*.

[31] Vasconcenlos L. H. C., Correia A. C. C., de Souza I. L. L. (2015). Flavonoid galetin 3,6-dimethyl ether attenuates Guinea pig ileum contraction through K^+^ channel activation and decrease in cytosolic calcium concentration. *European Journal of Pharmacology*.

[32] Gerthoffer W. T. (2005). Signal-Transduction Pathways that Regulate Visceral Smooth Muscle Function III. Coupling of muscarinic receptors to signaling kinases and effector proteins in gastrointestinal smooth muscles. *American Journal of Physiology-Gastrointestinal and Liver Physiology*.

[33] Bornstein J. C., Costa M., Grider J. R. (2004). Enteric motor and interneuronal circuits controlling motility. *Neurogastroenterology and Motility*.

[34] Yamaji M., Ohta M., Yamazaki Y. (2002). A possible role of neurotensin in NANC relaxation of longitudinal muscle of the jejunum and ileum of Wistar rats. *British Journal of Pharmacology*.

[35] Terauchi A., Kobayashi D., Mashimo H. (2005). Distinct roles of nitric oxide synthases and interstitial cells of Cajal in rectoanal relaxation. *American Journal of Physiology-Gastrointestinal and Liver Physiology*.

[36] Imran I., Hussain L., Zia-Ul-Haq M., Janbaz K. H., Gilani A. H., De Feo V. (2011). Gastrointestial and respiratory activities of Acacia leucophloea. *Journal of Ethnopharmacology*.

[37] Bello R., Moreno L., Primo-Yúfera E., Esplugues J. (2002). Globularia alypumL. extracts reduced histamine and serotonin contractionin vitro. *Phytotherapy Research*.

[38] Oliveira E. J., Romero M. A., Silva M. S., Silva B. A., Medeiros I. A. (2001). Intracellular calcium mobilization as a target for the spasmolytic action of scopoletin. *Planta Medica*.

[39] González-Trujano M. E., Gutiérrez-Valentino C., Hernández-Arámburo M. Y., Díaz-Reval M. I., Pellicer F. (2019). Identification of some bioactive metabolites and inhibitory receptors in the antinociceptive activity of *Tagetes lucida* Cav. *Life Science*.

[40] Ventura-Martínez R., Ángeles-López G. E., Rodríguez R., González-Trujano M. E., Déciga-Campos M. (2018). Spasmolytic effect of aqueous extract of *Tagetes erecta L*. flowers is mediated through calcium channel blockade on the Guinea-pig ileum. *Biomedicine & Pharmacotherapy*.

[41] Fanning M. J., Macander P., Drzewiecki G., Middleton E. (1983). Quercetin inhibits anaphylactic contraction of Guinea pig ileum smooth muscle. *International Archives of Allergy and Immunology*.

[42] Roghani M., Baluchnejadmojaradb T., Dehkordic F. (2006). The involvement of L-type voltage operated calcium channels in the vascular effect of quercetin in male rats. *Iranian Journal of Pharmaceutical Research*.

[43] Sandhu B. K., Tripp J. H., Candy D. C., Harries J. T. (1981). Loperamide: studies on its mechanism of action. *Gut*.

